# Mortalidade por Cardiomiopatias no Brasil

**DOI:** 10.36660/abc.20260438

**Published:** 2026-07-28

**Authors:** André Rodrigues Durães

**Affiliations:** 1 Universidade Federal da Bahia Hospital das Clínicas Salvador BA Brasil Hospital das Clínicas – Universidade Federal da Bahia, Salvador, BA – Brasil

**Keywords:** Cardiomiopatias, Mortalidade, Brasil

As cardiomiopatias representam um grupo heterogêneo de doenças do músculo cardíaco que, embora individualmente menos prevalentes que a doença arterial coronariana, impõem uma carga substancial de morbimortalidade em escala global. Dados do *Global Burden of Disease* (GBD) 2021 estimam que as cardiomiopatias foram responsáveis por aproximadamente 1,2 milhão de mortes no mundo, com uma taxa de mortalidade padronizada por idade de 14,3 por 100.000 habitantes, e mais de 28 milhões de anos de vida ajustados por incapacidade (DALYs) perdidos anualmente.^1,2^ A despeito dos avanços terapêuticos das últimas décadas, a trajetória epidemiológica dessas condições permanece marcada por profundas desigualdades regionais, tanto entre países em diferentes continentes, quanto dentro de um mesmo território nacional. É neste cenário que o estudo de Silva et al.,^3^ oferece uma contribuição oportuna e necessária ao analisar o perfil epidemiológico, além da evolução têmporo-espacial da mortalidade por cardiomiopatias no Brasil entre 2001 e 2021.

Trata-se de um importante estudo epidemiológico ecológico que utilizou dados do Sistema de Informações sobre Mortalidade (SIM) para examinar mais de 200.000 óbitos por cardiomiopatias no período de duas décadas. Observou-se que a mortalidade média foi de 6,66 por 100 mil habitantes, atingindo 7,64 por 100 mil em 2004 e caindo para 4,15 por 100 mil em 2020. Observou-se redução anual significativa de 1,86% (p<0,05) e a análise espacial revelou aglomerados de alta mortalidade principalmente no Centro-Oeste, Sul e Sudeste.^3^

Somado a estes dados interessantes, o dado mais impactante e relevante — uma redução média de 1,86% ao ano na taxa de mortalidade padronizada — é, em primeira análise, encorajador. Esse declínio posiciona o Brasil em trajetória semelhante à observada em países de renda média-alta, onde a melhoria no acesso ao diagnóstico e ao tratamento tem contribuído para a redução da mortalidade por doenças cardiovasculares não isquêmicas.^4^ No entanto, quando comparado à redução global estimada pelo GBD de aproximadamente 2,3% ao ano para o mesmo período,^1^ o ritmo brasileiro se revela aquém do desejável, sugerindo que os ganhos obtidos não foram uniformes nem suficientes para eliminar as disparidades internas.

A heterogeneidade espacial documentada é outro dado notável deste estudo. Enquanto as regiões Sul e Sudeste apresentaram declínios mais acentuados, com reduções anuais superiores a 2,5% em alguns estados, as regiões Norte e Nordeste exibiram tendências menos favoráveis, com taxas de mortalidade persistentemente mais elevadas e, em algumas áreas, sinais de estabilização ou mesmo aumento. Esse padrão não é idiossincrático do Brasil. Dados do GBD revelam que, globalmente, a carga de cardiomiopatias é desproporcionalmente concentrada em países de baixa e média renda, que respondem por mais de 80% dos DALYs perdidos.^2^ Por exemplo, na África Subsaariana e no Sul da Ásia, as taxas de mortalidade por cardiomiopatias chegam a ser três a quatro vezes superiores às observadas na Europa Ocidental e na América do Norte.^1,5^ O Brasil, com sua vasta extensão territorial e profundas desigualdades socioeconômicas, reproduz em escala nacional o que se observa no plano global: a carga da doença segue o gradiente da pobreza.

Os determinantes desta heterogeneidade são múltiplos e interligados. O estudo de Silva et al.^3^ aponta para a associação entre maior mortalidade e piores indicadores sociodemográficos, como menor escolaridade e maior proporção de população autodeclarada preta ou parda. Esses achados ecoam evidências robustas da literatura internacional. No Estudo Multicêntrico de Intervenção em Miocardite e Cardiomiopatia Aguda (IMAC2), McNamara et al.^6^ demonstraram que pacientes com cardiomiopatia dilatada de origem não isquêmica provenientes de comunidades socioeconomicamente desfavorecidas apresentam risco 40% maior de hospitalização por insuficiência cardíaca, independentemente de fatores clínicos e terapêuticos. Outra análise com metodologia similar evidenciou que a mortalidade por cardiomiopatia periparto é significativamente maior entre mulheres negras nos Estados Unidos, refletindo não apenas diferenças biológicas, mas, sobretudo, barreiras estruturais e socioeconômicas no acesso ao cuidado.^7^

No contexto brasileiro, a questão racial e socioeconômica adquire contornos particularmente complexos. A população miscigenada de não brancos (pretos, pardos, mulatos), que constitui a maioria nas regiões Norte e Nordeste (região economicamente mais pobre com piores indicadores), enfrenta não apenas menor acesso a serviços de saúde especializados, mas também maior exposição a fatores de risco modificáveis, como hipertensão arterial não controlada, diabetes mellitus e doença de Chagas — esta última, uma causa relevante de cardiomiopatia no Brasil, com distribuição geográfica que se sobrepõe às áreas de maior vulnerabilidade social.^8^ A interação entre determinantes sociais, etiologias infecciosas e acesso desigual ao cuidado cria um ciclo de perpetuação da doença que políticas de saúde fragmentadas dificilmente conseguem romper.

As implicações clínicas e de saúde pública são profundas. Do ponto de vista da prática médica, o reconhecimento de que a mortalidade por cardiomiopatias não se distribui aleatoriamente no território nacional impõe a necessidade de estratégias diagnósticas e terapêuticas regionalizadas. A incorporação de ecocardiografia *point-of-care* em unidades básicas de saúde de regiões remotas, a capacitação de médicos da atenção primária para o reconhecimento precoce de sinais de insuficiência cardíaca, e a criação de linhas de cuidado integradas entre atenção primária e serviços especializados, são medidas factíveis e com potencial de impacto significativo.^9^ Um ensaio clínico realizado na Índia por Agarwal et al.^10^ demonstrou que programas de cuidado coordenado para insuficiência cardíaca em comunidades de baixa renda reduziram a mortalidade em 25% em dois anos.

No plano das políticas públicas, o estudo de Silva et al.^3^ oferece um mapa claro das prioridades. As regiões Norte e Nordeste, assim como áreas específicas do Centro-Oeste e do interior do Nordeste, demandam investimentos direcionados em infraestrutura diagnóstica, oferta de medicamentos essenciais e fortalecimento da atenção especializada. A experiência internacional, documentada em análises sobre sistemas de saúde universais, sugere que a redução das desigualdades em saúde cardiovascular requer não apenas aumento de recursos, mas redesenho dos modelos de atenção para garantir que os serviços cheguem a quem mais precisa.^11,12^

Olhando para o futuro, algumas questões emergem como prioridades de pesquisa e ação. Primeiro, é necessário compreender em maior profundidade os determinantes locais da heterogeneidade observada — estudos ecológicos identificam associações, mas não estabelecem causalidade. Investigações com desenhos multinível, que integrem dados individuais e contextuais, são essenciais para orientar intervenções precisas. Segundo, a vigilância epidemiológica das cardiomiopatias no Brasil precisa ser aprimorada, com a incorporação de registros clínicos que permitam distinguir subtipos etiológicos — dilatada, hipertrófica, restritiva, periparto, chagásica — cujas trajetórias e respostas ao tratamento diferem substancialmente.^13^ Terceiro, é imperativo que as políticas de saúde incorporem explicitamente o princípio da equidade, alocando recursos de forma proporcional à necessidade, e não apenas à demanda. Quarto, a atuação enérgica e efetiva no controle dos fatores de risco modificáveis e condições crônicas cujo tratamento trazem grande impacto na redução de eventos cardiovasculares maiores, como hipertensão arterial, diabetes, dislipidemia, sedentarismo, obesidade, tabagismo, etilismo etc.

O estudo de Silva et al.^3^ não é apenas mais uma análise epidemiológica. É um chamado à ação. Em um país de dimensões continentais e desigualdades históricas, a constatação de que a mortalidade por cardiomiopatias está diminuindo, mas de forma desigual, deve servir como alerta e como roteiro. A redução de 1,86% ao ano é insuficiente quando contrastada com o potencial de prevenção e tratamento que a medicina cardiovascular oferece na atualidade. A heterogeneidade espacial documentada é inaceitável em um sistema de saúde que tem a universalidade e a equidade como princípios constitucionais. Recentemente o Brasil se enquadrou na categoria de desenvolvimento humano "muito alto" pelo IDHM (Índice de Desenvolvimento Humano Municipal) de 0,805 através da análise de três dimensões: longevidade (saúde), acesso ao conhecimento (educação) e padrão de vida (renda). Contudo, a conhecida universalização do acesso, embora exitosa em estabelecer um piso elevado de longevidade, encontra limites na capacidade de equalizar desfechos em condições que demandam cuidado especializado contínuo, como as cardiomiopatias.^14^

Cabe agora à comunidade científica, aos gestores de saúde e aos formuladores de políticas públicas transformar os dados deste estudo em ação concreta. As cardiomiopatias no Brasil têm nome, endereço e determinantes sociais. Ignorá-los não é apenas um erro científico — é uma falha ética.
