# Supplementary material for: Mortalidade por Cardiomiopatias no Brasil
Source: Arq Bras Cardiol. 2026 Jul 28;123(6):e20260438. [Article in Portuguese] doi: 10.36660/abc.20260438 (PMC13668442; doi:10.36660/abc.20260438)
Supplement: Translation

# Translation: Mortality from Cardiomyopathies in Brazil

**DOI:** 10.36660/abc.20260438i

**Authors:** André Rodrigues Durães

**Affiliations:** 1 Salvador BA Brasil Hospital das Clínicas – Universidade Federal da Bahia, Salvador, BA – Brasil

**Keywords:** Cardiomyopathies, Mortality, Brazil

Cardiomyopathies represent a heterogeneous group of heart muscle diseases that, although individually less prevalent than coronary artery disease, impose a substantial burden of morbidity and mortality on a global scale. Data from the Global Burden of Disease (GBD) 2021 estimate that cardiomyopathies were responsible for approximately 1.2 million deaths worldwide, with an age-standardized mortality rate of 14.3 per 100,000 inhabitants, and more than 28 million disability-adjusted life years (DALYs) lost annually.^1,2^ Despite therapeutic advances in recent decades, the epidemiological trajectory of these conditions remains marked by profound regional inequalities, both between countries on different continents and within the same national territory. It is in this context that the study by Silva et al.^3^ offers a timely and necessary contribution by analyzing the epidemiological profile, as well as the spatiotemporal evolution of mortality from cardiomyopathies in Brazil between 2001 and 2021.

This is an important ecological epidemiological study that used data from the Mortality Information System (SIM) to examine more than 200,000 deaths from cardiomyopathies over a two-decade period. The average mortality rate was observed to be 6.66 per 100,000 inhabitants, reaching 7.64 per 100,000 in 2004 and falling to 4.15 per 100,000 in 2020. A significant annual reduction of 1.86% (p<0.05) was observed, and spatial analysis revealed clusters of high mortality, mainly in the Central-West, South, and Southeast regions.^3^

In addition to these interesting data, the most impactful and relevant piece of information—an average annual reduction of 1.86% in the standardized mortality rate—is, at first glance, encouraging. This decline places Brazil on a trajectory similar to that observed in upper-middle-income countries, where improved access to diagnosis and treatment has contributed to a reduction in mortality from non-ischemic cardiovascular diseases.^4^ However, when compared to the global reduction estimated by the GBD of approximately 2.3% per year for the same period,^1^ the Brazilian pace falls short of what is desirable, suggesting that the gains achieved were neither uniform nor sufficient to eliminate internal disparities.

The documented spatial heterogeneity is another notable finding of this study. While the South and Southeast regions showed more pronounced declines, with annual reductions exceeding 2.5% in some states, the North and Northeast regions exhibited less favorable trends, with persistently higher mortality rates and, in some areas, signs of stabilization or even increase. This pattern is not unique to Brazil. GBD data reveal that, globally, the burden of cardiomyopathies is disproportionately concentrated in low- and middle-income countries, which account for more than 80% of missed DALYs.^2^ For example, in Sub-Saharan Africa and South Asia, mortality rates from cardiomyopathies are three to four times higher than those observed in Western Europe and North America.^1,5^ Brazil, with its vast territorial extension and profound socioeconomic inequalities, reproduces on a national scale what is observed globally: the burden of disease follows the gradient of poverty.

The determinants of this heterogeneity are multiple and interconnected. The study by Silva et al.^3^ points to an association between higher mortality and worse sociodemographic indicators, such as lower education and a higher proportion of the population self-identifying as Black or mixed-race. These findings echo robust evidence from the international literature. In the Multicenter Intervention Study on Myocarditis and Acute Cardiomyopathy (IMAC2), McNamara et al.^6^ demonstrated that patients with non-ischemic dilated cardiomyopathy from socioeconomically disadvantaged communities have a 40% higher risk of hospitalization for heart failure, regardless of clinical and therapeutic factors. Another analysis with a similar methodology showed that mortality from peripartum cardiomyopathy is significantly higher among Black women in the United States, reflecting not only biological differences, but, above all, structural and socioeconomic barriers to access to care.^7^

In the Brazilian context, the racial and socioeconomic issues take on particularly complex dimensions. The mixed-race population of non-whites (blacks, browns, mulattos), which constitutes the majority in the North and Northeast regions (the poorest region with the worst indicators), faces not only less access to specialized health services, but also greater exposure to modifiable risk factors, such as uncontrolled hypertension, diabetes mellitus, and Chagas disease—the latter being a significant cause of cardiomyopathy in Brazil, with a geographic distribution that overlaps with areas of greater social vulnerability.^8^ The interaction between social determinants, infectious etiologies, and unequal access to care creates a cycle of disease perpetuation that fragmented health policies can hardly break.

The clinical and public health implications are profound. From a medical practice standpoint, the recognition that mortality from cardiomyopathies is not randomly distributed across the country necessitates regionalized diagnostic and therapeutic strategies. The incorporation of point-of-care echocardiography in primary healthcare units in remote areas, the training of primary care physicians for the early recognition of signs of heart failure, and the creation of integrated care pathways between primary care and specialized services are feasible measures with the potential for significant impact.^9^ A clinical trial conducted in India by Agarwal et al.^10^ demonstrated that coordinated care programs for heart failure in low-income communities reduced mortality by 25% in two years.

In terms of public policy, the study by Silva et al.^3^ offers a clear map of priorities. The North and Northeast regions, as well as specific areas of the Central-West and the interior of the Northeast, require targeted investments in diagnostic infrastructure, the supply of essential medicines, and the strengthening of specialized care. International experience, documented in analyses of universal health systems, suggests that reducing inequalities in cardiovascular health requires not only increased resources but also a redesign of care models to ensure that services reach those who need them most.^11,12^

Looking to the future, several issues emerge as research and action priorities. First, it is necessary to understand in greater depth the local determinants of the observed heterogeneity—ecological studies identify associations, but do not establish causality. Investigations with multilevel designs, integrating individual and contextual data, are essential to guide precise interventions. Second, the epidemiological surveillance of cardiomyopathies in Brazil needs to be improved, with the incorporation of clinical records that allow distinguishing etiological subtypes—dilated, hypertrophic, restrictive, peripartum, Chagasic—whose trajectories and responses to treatment differ substantially.^13^ Third, it is imperative that health policies explicitly incorporate the principle of equity, allocating resources proportionally to need, and not just to demand. Fourth, energetic and effective action is needed in controlling modifiable risk factors and chronic conditions whose treatment has a major impact on reducing major cardiovascular events, such as hypertension, diabetes, dyslipidemia, sedentary lifestyle, obesity, smoking, alcoholism, etc.

The study by Silva et al.^3^ is not just another epidemiological analysis. It is a call to action. In a country of continental dimensions and historical inequalities, the finding that mortality from cardiomyopathies is decreasing, but unevenly, should serve as a warning and a roadmap. The 1.86% annual reduction is insufficient when contrasted with the potential for prevention and treatment that cardiovascular medicine currently offers. The documented spatial heterogeneity is unacceptable in a health system that has universality and equity as constitutional principles. Recently, Brazil was classified in the "very high" human development category by the MHDI (Municipal Human Development Index) of 0.805 through the analysis of three dimensions: longevity (health), access to knowledge (education), and standard of living (income). However, the well-known universalization of access, while successful in establishing a high level of longevity, has limitations in its ability to equalize outcomes in conditions that require continuous specialized care, such as cardiomyopathies.^14^

It is now up to the scientific community, health managers, and public policy makers to transform the data from this study into concrete action. Cardiomyopathies in Brazil have names, addresses, and social determinants. Ignoring them is not just a scientific error—it is an ethical failure.
